# Effects of a polyphenol-rich grape and blueberry extract (Memophenol™) on cognitive function in older adults with mild cognitive impairment: A randomized, double-blind, placebo-controlled study

**DOI:** 10.3389/fpsyg.2023.1144231

**Published:** 2023-03-29

**Authors:** Adrian L. Lopresti, Stephen J. Smith, Camille Pouchieu, Line Pourtau, David Gaudout, Véronique Pallet, Peter D. Drummond

**Affiliations:** ^1^Clinical Research Australia, Perth, WA, Australia; ^2^Healthy Ageing Research Centre and Discipline of Psychology, College of Science, Health, Engineering and Education, Murdoch University, Perth, WA, Australia; ^3^Activ'Inside, Beychac-et-Caillau, France; ^4^Université de Bordeaux, INRAE, Bordeaux INP, NutriNeurO, UMR, Bordeaux, France

**Keywords:** blueberries, grapes, mild cognition impairment, memory, polyphenols

## Abstract

**Background:**

Polyphenols are naturally occurring organic compounds found in plants. Research suggests that their intake reduces the risk of cognitive decline and related dementias. Grapes and blueberries are polyphenol-rich foods that have attracted attention for their potential cognitive-enhancing effects.

**Purpose:**

Examine the effects of supplementation with a standardized and patented polyphenol-rich grape and blueberry extract (Memophenol™) on cognitive function in older adults with mild cognitive impairment.

**Study design:**

Two-arm, 6 month, parallel-group, randomized, double-blind, placebo-controlled trial.

**Methods:**

One hundred and forty-three volunteers aged 60 to 80 years with mild cognitive impairment were supplemented with either 150 mg of Memophenol™, twice daily or a placebo. Outcome measures included computer-based cognitive tasks, the Behavior Rating Inventory of Executive Function (BRIEF-A), the Cognitive Failures Questionnaire, and the CASP-19.

**Results:**

Compared to the placebo, Memophenol™ supplementation was associated with greater improvements in the speed of information processing (*p* = 0.020), visuospatial learning (*p* = 0.012), and the BRIEF-A global score (*p* = 0.046). However, there were no other statistically significant between-group differences in the performance of other assessed cognitive tests or self-report questionnaires. Memophenol™ supplementation was well-tolerated with no reports of significant adverse reactions.

**Conclusion:**

The promising results from this trial suggest that 6-months of supplementation with Memophenol™ may improve aspects of cognitive function in adults with mild cognitive impairment. Further research will be important to expand on the current findings and identify the potential mechanisms of action associated with the intake of this polyphenol-rich extract.

## Introduction

There is increasing evidence of a relationship between diet and cognitive health. In a meta-analysis based on data from over 34,000 participants, people consuming a high Mediterranean-based diet had an approximate 20% reduced risk of developing a cognitive disorder compared to people eating a low Mediterranean diet (Wu and Sun, 2017). Food characteristic of a traditional Mediterranean diet include fruits, vegetables, whole grains, legumes, nuts, seeds, and extra-virgin olive oil. Such a diet is characterized by a high intake of phenolic-rich foods. Polyphenols are a large family of naturally occurring organic compounds found in plants, and results from epidemiological studies have confirmed that their intake is associated with a reduced risk of cognitive decline. Based on data from 2,801 participants followed for an average of 19.7 years, individuals with the highest (>60th percentile) intake of various flavonoids (a class of polyphenols) had a significantly lower risk of Alzheimer’s disease (AD) and related dementias relative to individuals with the lowest intakes (≤15th percentile) (Shishtar et al., 2020). In a systematic review and meta-analysis based on data from 44 randomized controlled trials, it was concluded that short-to-moderate-term polyphenol interventions might improve working and episodic memory in middle-to-older-aged adults; however, publication bias impacted on the robustness of conclusions (De Vries et al., 2021). In another review based on 28 epidemiological studies (8 cross-sectional and 20 cohort studies) and 55 randomized trials, it was concluded that there was preliminary evidence to suggest a beneficial effect of polyphenol intake on cognitive function (Yang et al., 2021). However, in a systematic review of 22 studies, it was concluded that the evidence was mixed and that further randomized-controlled trials were necessary before definitive conclusions could be made (Colizzi, 2019).

Two commonly consumed polyphenol-rich foods include grapes and blueberries, both of which have attracted interest as foods that may enhance cognitive performance and protect against cognitive decline. In a systematic review of eight studies, preliminary results suggested that grapes improved some aspects of cognition (eg, executive function, processing speed, and spatial memory) after chronic interventions, although differences in study designs, dosages, and outcome measures impacted the strength of conclusions (Bird et al., 2022). Moreover, in a systematic review based on 7 adult clinical studies, it was concluded that blueberry supplementation may enhance delayed memory, executive function, and psychomotor function in healthy older-age adults and adults with mild cognitive impairment (MCI) (Hein et al., 2019). The majority of these studies have examined their cognitive effects when these foods were delivered in isolation. However, in an animal trial, the synergistic effects of these two ingredients were indicated as demonstrated by a 3-to-5-fold increase in plasma concentrations of blueberry phenolic metabolites along with an equivalent decrease in their appearance in feces when they were co-ingested compared to their delivery in isolation (Dudonne et al., 2016). There are several speculated mechanisms associated with the potential neuroprotective effects of blueberries and grapes. Findings from preclinical and *in vitro* trials suggest that blueberries and grapes may reverse or prevent neuronal aging by reducing oxidative stress (Joseph et al., 1999), upregulating neuronal signaling proteins (Williams et al., 2008; Rendeiro et al., 2012), increasing the viability and proliferation of adult hippocampal human neural progenitor cells (Zheng et al., 2022), reducing neuroinflammation (Shukitt-Hale et al., 2008), and protecting against amyloid β-induced cytotoxicity (Jeong et al., 2013). In a study on adult mice, the administration of a polyphenol-rich extract from grapes and berries attenuated cognitive decline and increased hippocampal nerve growth factor mRNA expression. The supplemented aged mice also displayed a greater proportion of newly generated neurons with prolongations than control age-matched mice (Bensalem et al., 2018).

The effects of a polyphenol-rich grape and blueberry extract (Memophonol™, 600 mg) on cognition have been examined in two clinical studies. Its acute administration in 30 healthy students aged between 18 and 25 years increased performance on a serial three-subtraction task compared to a placebo, and there was a trend toward improved performance on a rapid visual information processing task (Philip et al., 2019). In a placebo-controlled study on healthy older-age adults aged 60 to 70 years, compared to the placebo, the 6-month intake of Memophenol™ improved visuospatial and verbal recognition memory (measures of episodic memory) in a subset of participants defined as “cognitive decliners.” Cognitive decliners were the quartile of participants with the lowest baseline performances on the paired associate learning task (Bensalem et al., 2019). This result suggests that its intake may have greater benefits for individuals experiencing some form of cognitive impairment. Consequently, the aims of this study were to examine the effects of a 24-week administration of Memophenol™ in older adults with MCI. As beneficial effects were observed in older-aged participants with more advanced cognitive decline, a lower and more cost-effective dose of 300 mg daily was trialed in this study in people with MCI. It was hypothesized that compared to a placebo, its administration would be associated with improvements in episodic memory and other cognitive domains as measured by computer-based cognitive tasks and self-report questionnaires.

## Materials and methods

### Study design

**Figure 1:**
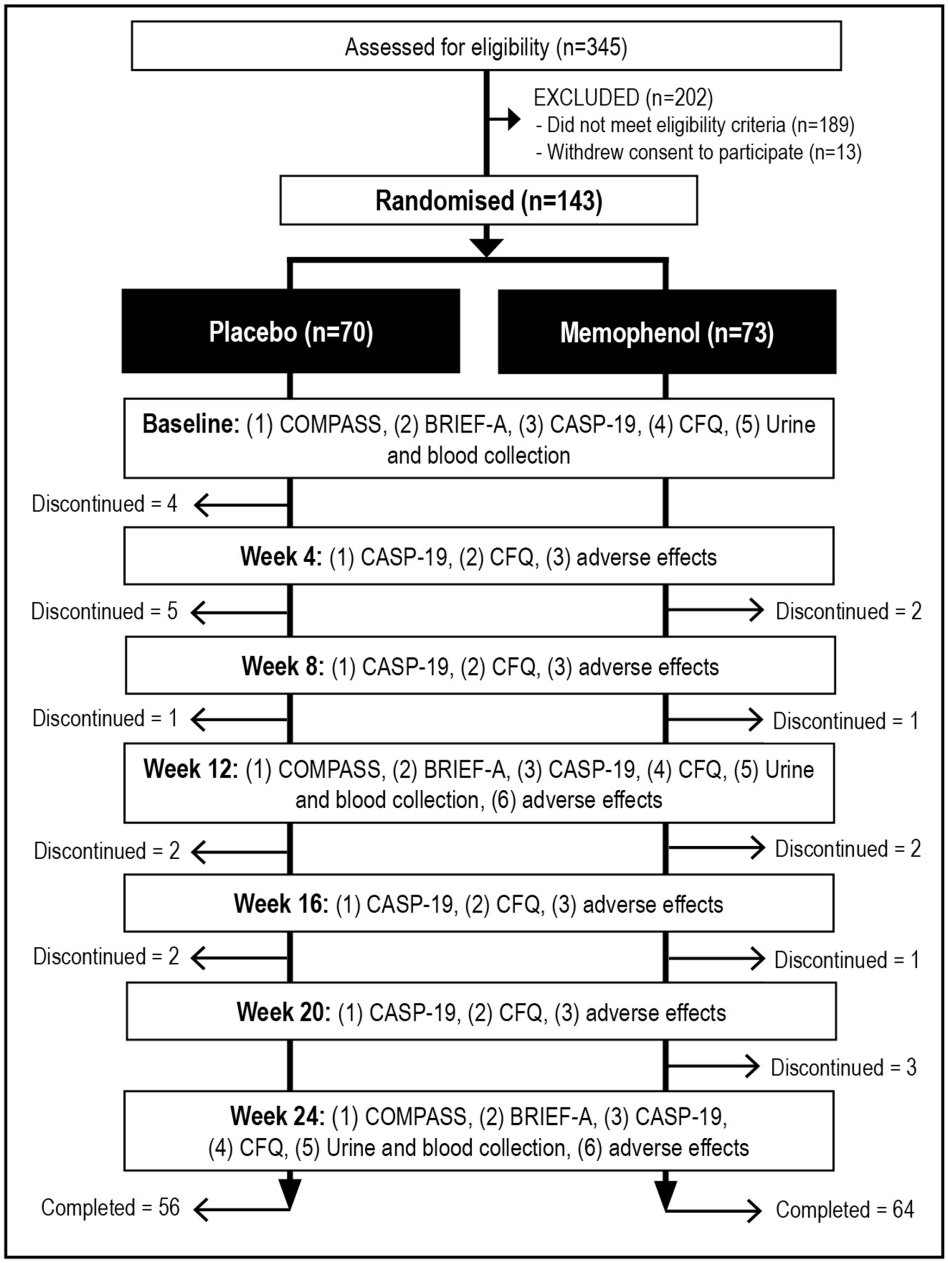
Systematic illustration of study design.

This was a 24 week, two-arm, parallel-group, randomized, double-blind, placebo-controlled trial (Figure 1). The study protocol was approved by the National Institute of Integrative Medicine Human Research Ethics Committee (approval number 0085E_2021), and informed consent was obtained from all participants. This trial was prospectively registered with the Australian and New Zealand Clinical Trials Registry (ACTRN12621000496819).

### Recruitment and randomization

Volunteers aged between 60 and 80 years were recruited between June and December 2021 through social media advertisements and email databases. Volunteers interested in the study visited a website page with information about the trial and a link to complete a screening questionnaire. This questionnaire assessed for self-reported memory problems; medication use; medical or psychiatric disorder history; alcohol, nicotine, and other drug use; and herbal and nutraceutical intake. To assess the severity of depressive and anxiety symptoms, respondents completed the 4-item Patient Health Questionnaire (PHQ-4) (Kroenke et al., 2009) and the Geriatric Depression Scale – Short Form (GDS-SF) (Van Marwijk et al., 1995). If assessed as likely eligible, a telephone interview was conducted where participants were asked questions to confirm their eligibility and to obtain further demographic information. This included the completion of the telephone-administered Montreal Cognitive Assessment–Blind Version (MoCA-BV), which is a validated and commonly used clinician-administered assessment for MCI (Wittich et al., 2010). The MoCA-BV contains the same items as the original MoCA, except questions requiring visual abilities have been removed. A score of 13 to 18 on the MoCA-BV falls within the MCI range.

Eligible and consenting participants were then allocated randomly to one of two groups (Memophenol™ or placebo; 1:1 ratio). To ensure the concealment of sequencing, a randomization calculator^1^ was used with the randomization structure involving 14 permuted blocks, with 10 participants per block. The randomization sequence was established by a researcher not directly involved in volunteer recruitment, and bottle codes were held by the study sponsor and revealed after all data were collected. Identification numbers were allocated to participants based on their order of enrolment in the study. All capsules were packaged in matching bottles. Study investigators were blind to the treatment group allocation until all outcome data were collected and a blind review was undertaken.

### Participants

#### Inclusion criteria

Male and female participants aged 60 to 80 years, experiencing self-reported difficulties with attention and memory, and scoring between 13 and 18 on the MoCA-BV were recruited. Participants were living independently, were non-smokers, had a body mass index (BMI) between 18 and 30 kg/m^2^, and had no plan to commence any new treatments during the study period. All participants consented to all pertinent aspects of the trial through the completion of an online consent form.

#### Exclusion criteria

Participants were considered ineligible if they had a diagnosis of dementia based on the revised National Institute on Aging-Alzheimer’s Association (NIA/AA) criteria; suffered from a recently diagnosed (within 3 months) or unmanaged medical condition including but not limited to hyper/hypotension, cardiovascular disease, diabetes, gallbladder disease, gastrointestinal disease requiring regular medication use, endocrine disease, autoimmune disease, cancer/malignancy, or acute or chronic pain condition; were diagnosed with a psychiatric disorder (other than mild-to-moderate depression or anxiety), or scored greater than 5 on the GDS-SF, or greater than 7 on the PHQ-4; were diagnosed with a neurological condition such as Alzheimer’s or Parkinson’s disease; had a history of intracranial hemorrhage, seizures, stroke, or a head injury (with loss of consciousness); regularly took medications including but not limited to blood thinners and anti-hypertensive drugs, anticholinergics, anticoagulants, acetylcholinesterase inhibitors, or steroid medications; had a change in medication in the past 90 days or expected to change during the study duration; reported nutrient deficiencies including low iron or vitamin B12; were taking vitamins, herbal, or omega-3 supplements that were expected to influence study outcomes; had fragile veins and past difficulty in giving blood; had a current or 12-month history of illicit drug abuse; consumed greater than 14 standard alcoholic drinks per week; had any major surgeries over the past year; or had planned significant lifestyle change in the next 6 months.

### Interventions

The intervention contained a standardized and patented polyphenol-rich blend of grape and wild blueberry extracts (Memophenol™, patent WO/2017/072219, Activ’Inside, Beychac et Caillau, France) or a placebo. Memophenol™ and placebo capsules were identical in appearance, matched for color, shape, size, smell, and taste. The intervention contained 150 mg of Memophenol™ per capsule and the placebo capsules contained maltodextrin. All volunteers were instructed to take 1 capsule, twice daily with food, for 24 weeks, delivering 300 mg daily of Memophenol™. Although it is difficult to accurately estimate, based on the total polyphenol content, this daily dose is equivalent to eating approximately 185 g of grapes (35 to 40 grapes) or 34 g of blueberries (65 to 70 blueberries) a day. Capsule adherence was evaluated by asking participants to estimate the consistency of capsule intake (0 to 100%) every 4 weeks and by the return of unused capsules at the week 12 and 24 assessments. The efficacy of treatment blinding was assessed by asking participants to guess group allocation (placebo, Memophenol™, or unsure) at the completion of the study.

### Outcome measures

#### Cognitive skills comprising episodic memory, working memory, accuracy of attention, speed of information processing, and visuospatial learning

The Computerized Mental Performance Assessment System (COMPASS) (Northumbria University, Newcastle upon Tyne, UK) was used for the computer-based cognitive testing. The results of the COMPASS have been demonstrated in several studies to be sensitive to nutritional and dietary interventions (Kennedy et al., 2017; Lopresti et al., 2021). Assessments were completed onsite at baseline, week 12, and week 24. Participants completed a brief practice run at the beginning of each assessment to familiarize themselves with the computer tasks and then completed the battery of cognitive tasks as detailed in Supplementary Table S1. Assessments were administered between 8 and 11 am after an overnight fast, with assessments for each participant occurring at approximately the same time. To control for the potential variability in the timing and quantity of caffeine intake across visits, participants were also asked to not consume any caffeinated beverage the morning of their assessment. They were also instructed to not consume alcohol the evening before testing. Episodic memory (primary outcome measure), working memory, accuracy of attention, and visuospatial learning were evaluated by calculating the mean percentage of the cognitive tasks detailed in Supplementary Table S2. The speed of information processing was assessed by calculating the mean reaction time of cognitive tasks detailed in Supplementary Table S2.

#### Behavior rating inventory of executive function-adult version

The BRIEF-A is a validated self-report questionnaire assessing executive function in adults aged 18 to 90 years. It contains 75 items where scores are calculated for an overall global score and two index scores comprising the behavioral regulation index and the metacognition index (Roth et al., 2005). The online version of the BRIEF-A was completed onsite at weeks 0, 12, and 24. Lower scores on the BRIEF-A indicate better executive function.

#### Cognitive failures questionnaire

The CFQ is a self-report 25-item questionnaire that assesses the frequency of cognitive difficulties (Broadbent et al., 1982). The CFQ has sound psychometric properties (Bridger et al., 2013), where lower scores indicate improved cognitive skills. An online version of the CFQ was completed at baseline and every 4 weeks thereafter. At baseline, week 12, and week 24, the CFQ was completed onsite, and the remaining administrations were completed at participants’ work or home environment.

#### Control, autonomy, self-realization, and pleasure (CASP-19)

The CASP-19 is a 19-item measure of well-being developed for older people (Hyde et al., 2003). Questions are rated on a 4-point scale ranging from never to often, with higher scores suggesting better well-being. In studies on older adults with dementia, and community-dwelling older-age adults, the CASP-19 had good psychometric properties (Sim et al., 2011; Stoner et al., 2019). An online version of the CASP-19 was administered at baseline and every 4 weeks thereafter. At baseline, week 12, and week 24, the CASP-19 was completed onsite, and the remaining administrations were completed at participants’ work or home environment.

#### Polyphenol food intake

To assess for polyphenol intake, participants completed a food diary for 3 days before their baseline, week 12, and week 24 assessments. Consumed foods and liquids were entered into Nutrilog,^2^ and the mean daily polyphenol intake was estimated based on the following food databases: Nuttab 2019 -Release 1, Mc Cance, Nuttab 2010, and USDA.

#### Adverse effects

The tolerability of capsule intake was assessed every 4 weeks through an online question enquiring about adverse effects that were believed to be due to capsule intake. Participants were also requested to contact researchers if they experienced any adverse effects.

### Sample size calculation

An *a priori* power analysis was undertaken to estimate the required sample size (based on a single outcome variable) and was powered based on episodic memory. Regarding flavonoids, a previous study assessing the efficacy of Memophenol™ on memory function identified a significant change in immediate free recall from the Verbal Recognition Memory task (CANTAB battery) in a subgroup of “decliners” after 24 weeks of supplementation (Bensalem et al., 2019). Cohen’s d was approximately 0.6 (medium effect size). Based on the results of this study, a power calculation with a medium effect size of 0.5 was used, which required a sample size of 64 per group for a two-arm trial, with an 80% power to detect a significant change at a two-sided 5% significance level. The recruited group sizes of 70 assumed a 10% attrition rate.

### Statistical analysis

For baseline data, Pearson’s Chi-square test was used to compare categorical data, an independent samples t-test was used to compare group data for normalized continuous variables, and the Mann–Whitney U-Test was used for non-normalized continuous data. Outcome analyses were conducted using intention-to-treat (ITT), with all participants retained in originally assigned groups. Generalized Linear Mixed Models (GLMM) assessed differences between intervention groups on primary, secondary, and exploratory outcomes over time, with intervention effects assessed *via* entry of the intervention group (placebo and Memophenol™) x time interaction. Time points considered for each measure comprised: (1) COMPASS measures (weeks 0, 12, and 24) comprising episodic memory (primary outcome measure), speed of information processing, accuracy of attention, (2) BRIEF-A global score (weeks 0, 12, and 24), (3) CASP-19 total score (weeks 0, 4, 8, 12, 16, 20, and 24), and (4) CFQ total score (weeks 0, 4, 8, 12, 16, 20, and 24). The COMPASS groupings are consistent with other studies that have administered the COMPASS as an outcome measure (Kennedy et al., 2019; Wightman et al., 2020). As an exploratory analysis, further analyses using GLMM were conducted on the BRIEF-A behavioral regulation index and metacognition index. Random intercepts were utilized in each GLMM model, and covariates of age, sex, baseline BMI, educational level, average energy intake, and average dietary polyphenol intake were included as fixed effects. Where applicable, gamma (with log link function) and normal (with identity link function) target distributions were used. Appropriate covariance structures were used to model correlation associated with repeated time measurements in gamma models. Robust estimations were used to handle any violations of model assumptions. Intervention group differences at time points were assessed using simple contrasts. As a further exploratory analysis, the effects of changes in dietary polyphenol intake (in milligrams) on changes in COMPASS cognitive domains (week 0 to 24) in participants in the Memophenol™ group was examined using a Pearson correlation coefficient (*r*). As a measure of visuospatial learning, a visit x trial x group, repeated-measures ANCOVA was performed on the displacement scores (trials 1 to 5) on the location learning task (covariates of age, sex, baseline BMI, educational level, average energy intake, and average dietary polyphenol intake). Moreover, a trial x group, repeated-measures ANCOVA was conducted at each visit (weeks 0, 12, and 24) to examine differences in visuospatial learning at each visit. Data from participants were included in the location learning analyses if data were obtained at week 12 [last observation carried forward from week 12 for missing values]. As the location learning displacement scores were not normally distributed, the data were winsorized, where scores greater than 3 standard deviations from the mean were replaced with the highest score that fell below the criterion of 3 standard deviations from the mean. All results were analyzed using SPSS (version 28; IBM, Armonk, NY) using a value of p of ≤0.05. Due to the exploratory nature of this trial, there was no adjustment to the value of *p* for multiple testing.

## Results

### Baseline questionnaire and demographic information

As detailed in Figure 1, from 345 people who completed the initial online screening questionnaire, 189 did not fulfill the eligibility criteria, and 13 withdrew consent to participate in the study. Fourteen people allocated to the placebo group withdrew from the study, and 9 people from the Memophenol™ group. Reasons for withdrawal in the placebo group included medical reasons unrelated to the study (*n* = 4), no reason given (*n* = 2), digestive symptoms (*n* = 2), skin rash (*n* = 1), starting another clinical trial (*n* = 1), worsening memory (*n* = 1), migraines (*n* = 1), heart palpitations (n = 1), and commencing a weight loss medication (*n* = 1). Reasons for withdrawal in the Memophenol™ group included family-related issues (*n* = 4), unexpected travel (*n* = 3), personal issues (*n* = 1), and excessive sleepiness (*n* = 1). Details of participant baseline scores and background information of the total sample are detailed in Table 1.

**Table 1:** Baseline sociodemographic and clinical characteristics.

		Memophenol™ (*n* = 73)	Placebo (*n* = 70)	value of *p*
Age	Mean	68.36	67.37	0.177^a^
	SE	0.54	0.62	
Sex	Male (*n*)	19 (26%)	20 (29%)	0.733^b^
	Female (*n*)	54 (74%)	50 (71%)	
BMI	Mean	26.27	26.52	0.404^a^
	SE	0.34	0.43	
Marital status	Single (*n*)	20 (27%)	19 (27%)	0.973^b^
	Married/ Defacto (*n*)	53 (73%)	51 (73%)	
Education	Secondary (*n*)	36 (60%)	44 (51%)	0.558^b^
	Tertiary (*n*)	23 (26%)	19 (33%)	
	Post-graduate (*n*)	11 (14%)	10 (16%)	
Blood Pressure -Systolic	Mean	139.95	134.47	0.021^c^
	SE	1.69	2.08	
Blood Pressure -Diastolic	Mean	84.10	84.04	0.487 ^c^
	SE	1.09	1.19	
IPAQ Category	Low (*n*)	32 (49%)	36 (46%)	0.789^b^
	Moderate (*n*)	30 (43%)	31 (43%)	
	High (*n*)	8 (8%)	6 (11%)	
PHQ-4	Mean	1.40	1.21	0.326^a^
	SE	0.21	0.23	
GDS	Mean	2.26	1.76	0.054 ^a^
	SE	0.18	0.18	
MoCA	Mean	16.71	16.27	0.071^a^
	SE	0.19	0.20	
Polyphenol intake (mg)	Mean	1994	2033	0.393^c^
	SE	113.15	84.12	
BRIEF-A: Behavioral Regulation Index	Mean	45.33	45.10	0.608^a^
	SE	0.95	1.08	
BRIEF-A: Metacognition Index	Mean	64.66	62.49	0.375^a^
	SE	1.48	1.36	
BRIEF-A: Global Score	Mean	109.99	107.59	0.401 ^a^
	SE	2.29	2.25	
CFQ	Mean	41.58	39.46	0.172^a^
	SE	1.36	1.38	
CASP	Mean	42.79	45.03	0.036^a^
	SE	0.82	0.86	

a = Independent-Samples Mann–Whitney U Test; b = Pearson Chi-Square Test; c = Independent-Samples T-Test.

### Outcome measures

#### Primary outcome measure

##### Episodic memory

As demonstrated in Table 2, based on the GLMM, there was no statistically significant time x group interaction in episodic memory over time (*p* = 0.777). In the Memophenol™ group, there was a statistically significant 3.99% improvement in episodic memory from baseline to week 24 (*p* < 0.001) and a statistically significant 3.36% improvement in the placebo group (*p* = 0.003).

**Table 2:** Change in cognitive performance (estimated marginal means).

		Memophenol™ (*n* = 64)				Placebo (*n* = 56)				value of *p*^b^
		Week 0	Week 12	Week 24	value of p^a^	Week 0	Week 12	Week 24	value of *p*^a^	
Episodic Memory (mean percentage on cognitive tasks)	Mean	65.91	67.44	68.54	< 0.001	65.49	67.25	67.69	0.003	0.777
	SE	0.70	0.66	0.69		0.62	0.79	0.85		
Working memory (mean percentage on cognitive tasks)	Mean	64.07	65.49	64.87	0.256	63.75	64.35	64.68	0.254	0.548
	SE	0.78	0.62	0.70		0.76	0.81	1.01		
Speed of information processing (mean reaction time on cognitive tasks)	Mean	977.89	921.56	884.72	< 0.001	919.40	907.13	889.39	0.023	0.023
	SE	24.46	20.43	15.34		17.13	17.26	18.85		
Accuracy of attention (mean percentage on cognitive tasks)	Mean	93.59	93.71	93.60	0.995	93.89	94.69	94.02	0.889	0.825
	SE	0.85	1.03	1.20		0.75	0.81	1.29		

Results (estimated means) are generated from generalized mixed-effects models adjusted for age, sex, BMI, educational level, dietary energy intake, and dietary polyphenol intake.

a *p*-values are generated from repeated measures generalized mixed-effects models adjusted for age, sex, BMI, educational level, dietary energy intake, and dietary polyphenol intake (time effects baseline, week 12, and 24).

b *p*-values are generated from repeated measures generalized mixed-effects models adjusted for age, sex, BMI, educational level, dietary energy intake, and dietary polyphenol intake (time x group interaction).

### Secondary outcome measures

#### Working memory

As demonstrated in Table 2, based on the GLMM, there was no statistically significant time x group interaction in working memory over time (*p* = 0.548). In both the Memophenol™ and placebo groups, there was no significant change in working memory from baseline to week 24.

#### Speed of information processing

As demonstrated in Table 2, based on the GLMM, there was a statistically significant time x group interaction in the speed of information processing over time (*p* = 0.023). In the Memophenol™ group, there was a statistically significant 9.53% reduction in reaction time from baseline to week 24 (*p* < 0.001) compared with a smaller but statistically significant 3.26% reduction in the placebo group (*p* = 0.023).

#### Accuracy of attention

As demonstrated in Table 2, based on the GLMM, there was no statistically significant time x group interaction in the accuracy of attention over time (*p* = 0.825). In both the Memophenol™ and placebo groups, there was no significant change in the accuracy of attention from baseline to week 24.

#### Visuospatial learning

**Figure 2:**
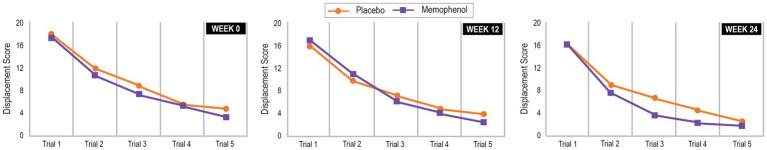
Location learning displacement scores at each visit.

In the location learning task (comprising 5 learning trials), a repeated-measures ANOVA visit x trial x group analysis revealed a statistically significant interaction (*p* = 0.029) (Table 3, Figure 2). An analysis of performance at the different visits revealed there was a statistically significant trial x group interaction at week 24 (*p* = 0.003). This indicates a better rate of learning in the Memophenol™ group compared to the placebo at week 24.

**Table 3:** Change in location learning displacement scores.

		Week 0						Week 12						Week 24						value of *p*^b^
		Trial 1	Trial 2	Trial 3	Trial 4	Trial 5	*p*-value^a^	Trial 1	Trial 2	Trial 3	Trial 4	Trial 5	*p*-value^a^	Trial 1	Trial 2	Trial 3	Trial 4	Trial 5	*p*-value^a^	
Memophenol™ (*n* = 69)	Mean	17.39	10.64	7.45	5.13	3.14	0.441	16.86	10.83	6.16	3.97	2.35	0.065	16.03	7.46	3.64	2.33	1.77	0.003	0.029
	SE	0.94	0.88	0.80	0.76	0.61		0.92	0.74	0.76	0.69	0.47		1.05	0.75	0.48	0.41	0.37		
Placebo (*n* = 60)	Mean	17.95	11.90	8.85	5.57	4.92		15.85	9.65	7.10	4.80	3.83		16.05	8.90	6.77	4.58	2.62		
	SE	1.04	0.98	0.86	0.79	0.80		1.00	0.97	0.96	0.83	0.74		1.15	1.00	0.96	0.78	0.52		

a Repeated-measures ANCOVA trial x group interaction *p*-value (covariates: age, sex, BMI, educational level, dietary energy intake, and dietary polyphenol intake).

b Repeated-measures ANCOVA visit x group x trial interaction value of *p* (covariates: age, sex, BMI, educational level, dietary energy intake, and dietary polyphenol intake).

#### BRIEF-A global score

As demonstrated in Table 4, based on the GLMM, there was a statistically significant time x group interaction in the BRIEF-A global score over time (*p* = 0.049). In the Memophenol™ group, there was a statistically significant 5.90% reduction in the global score from baseline to week 24 (*p* < 0.001) compared to a non-significant 1.89% reduction in the placebo group (*p* = 0.141).

**Table 4:** Change in self-report questionnaires (estimated marginal means).

		Memophenol™ (*n* = 64)								Placebo (*n* = 56)								*p*-value
		Week 0	Week 4	Week 8	Week 12	Week 16	Week 20	Week 24	*p*-value	Week 0	Week 4	Week 8	Week 12	Week 16	Week 20	Week 24	*p*-value	
BRIEF-A -Global Score	Mean	109.42	–	–	106.99	–	–	102.96	< 0.001	106.41	–	–	104.31	–	–	104.40	0.141	0.049
	SE	2.75	–	–	2.87	–	–	2.77		2.37	–	–	2.33	–	–	2.55		
BRIEF-A -Metacognitive Index	Mean	64.86	–	–	63.46	–	–	60.71	< 0.001	62.14	–	–	60.78	–	–	61.08	0.225	0.026
	SE	1.77	–	–	1.87	–	–	1.74		1.44	–	–	1.40	–	–	1.52		
BRIEF-A -Behavioral regulation index	Mean	44.55	–	–	43.51	–	–	42.23	0.001	44.25	–	–	43.50	–	–	43.29	0.162	0.294
	SE	1.12	–	–	1.20	–	–	1.16		1.10	–	–	1.13	–	–	1.20		
CFQ total score	Mean	40.25	37.35	34.52	37.04	32.82	32.43	34.07	< 0.001	38.28	35.26	32.80	35.58	32.47	32.16	34.85	0.003	0.412
	SE	1.61	1.52	1.60	1.60	1.56	1.62	1.69		1.43	1.45	1.48	1.40	1.47	1.53	1.64		
CASP score	Mean	43.55	43.48	43.17	43.76	43.86	43.25	43.91	0.589	45.48	44.68	44.56	46.18	44.97	45.30	45.32	0.765	0.538
	SE	0.91	0.93	0.98	0.94	0.98	1.08	1.03		0.93	1.04	1.15	1.10	1.07	1.12	1.06		

Results (estimated means) are generated from generalized mixed-effects models adjusted for age, sex, BMI, educational level, dietary energy intake, and dietary polyphenol intake.^a^*p*-values are generated from repeated measures generalized mixed-effects models adjusted for age, sex, BMI, educational level, dietary energy intake, and dietary polyphenol intake.^b^*p*-values are generated from repeated measures generalized mixed-effects models adjusted for age, sex, BMI, educational level, dietary energy intake, and dietary polyphenol intake (time x group interaction).

#### Cognitive failures questionnaire

As demonstrated in Table 4, based on the GLMM, there was no statistically significant time x group interaction in CFQ scores over time (*p* = 0.412). In both the Memophenol™ (*p* < 0.001) and placebo (*p* = 0.003) groups, there were statistically significant reductions in CFQ scores from baseline to week 24.

#### Control, autonomy, self-realization, and pleasure (CASP-19)

As demonstrated in Table 4, based on the GLMM, there was no statistically significant time x group interaction in CFQ scores over time (*p* = 0.538). In both the Memophenol™ (*p* = 0.589) and placebo (*p* = 0.765) groups, there were no statistically significant reductions in CASP-19 scores from baseline to week 24.

### Exploratory outcome measures

#### COMPASS subscale scores

As demonstrated in Supplementary Table S3, based on the GLMM, there was a statistically significant time x group interaction in the reaction time on the picture recognition task over time (*p* = 0.009). In the Memophenol™ group, there was a statistically significant 4.96% reduction in reaction time from baseline to week 24 (*p* = 0.004) compared to a non-significant increase of 3.33% in the placebo group (*p* = 0.153). There were no other statistically significant time x group interactions on other COMPASS cognitive tasks.

#### BRIEF-A subscale scores

As demonstrated in Table 4, based on the GLMM, there was a statistically significant time x group interaction in the BRIEF-A metacognition index (*p* = 0.026) but not the behavioral regulation index (*p* = 0.294). In the Memophenol™ group, there was a statistically significant 6.40% reduction in the metacognition index score from baseline to week 24 (*p* < 0.001) compared to a non-significant 1.71% reduction in the placebo group (*p* = 0.225).

#### Polyphenol dietary intake and cognitive performance

In the Memophenol™ group, a correlational analysis revealed that change in dietary polyphenol intake from baseline to week 24 was not associated with changes in episodic memory (*r* = −0.106, *p* = 0.409), working memory (*r* = 0.096, *p* = 0.452), speed of information processing (*r* = −0.017, *p* = 0.895), or accuracy of attention (*r* = −0.124, *p* = 0.334). This suggests that dietary polyphenol intake had no effect on the cognitive-enhancing efficacy of Memophenol™ over time.

### Intake of supplements

Capsule bottles with remaining capsules were returned at the week-24 assessment, and participants completed a daily medication monitoring phone application. Based on these details, 97% of participants who completed the study took more than 80% of their capsules.

### Dietary changes

An analysis of dietary changes over time, based on the 3-day food records, indicated that there were no significant time x group differences in energy (kilocalories) (*p* = 0.343) or polyphenol (mg) intake (*p* = 0.809).

### Efficacy of participant blinding

To assess the effectiveness of condition concealment during the study, participants predicted at the end of the trial their condition allocation (i.e., placebo, Memophenol™, or unsure). Overall group concealment was high as 63% of participants either were unsure or incorrectly guessed treatment allocation.

### Adverse reactions

The frequency of self-reported adverse reactions is included in Table 5. No serious adverse reactions were reported by participants, and a similar frequency of adverse reactions was reported in both groups. Five participants withdrew from the trial due to a moderate severity of self-reported adverse reactions associated with capsule intake. One person in the Memophenol™ group withdrew due to increased sleepiness and 4 people withdrew from the study in the placebo group due to self-reported mild digestive complaints (*n* = 2), skin rash (*n* = 1), migraines (*n* = 1), and heart palpitations (*n* = 1). There were no accounts of any adverse reactions in 78% of participants in the Memophenol™ group and 84% in the placebo group. Further analyses revealed there were no differences between the groups in changes in BMI (*p* = 0.286), systolic blood pressure (*p* = 0.132), and diastolic blood pressure (*p* = 0.215) over time.

**Table 5:** Frequency of self-reported adverse events.

	Placebo	Memophenol™
Heart palpitations	0	1
Headaches	0	2
Dizziness/ confusion	1	2
Diarrhea	0	2
Constipation	2	0
Nausea	2	3
Heartburn/reflux	1	1
Stomach cramps	2	1
Worsened memory	1	0
Tiredness	0	4
Weight gain	0	2
Increased appetite	0	1
Metallic/plastic taste in the mouth	1	1
Skin rash/ Itchy skin	2	0
Lightheaded	1	0
Total number of adverse effects	13	20

## Discussion

In this 24 week, randomized, double-blind, placebo-controlled trial on adults aged 60 to 80 years with MCI, the administration of 300 mg daily of a blueberry and grape extract (Memophenol™) was associated with significantly greater improvements in the speed of information processing and visuospatial learning compared with the placebo. However, there were no significant between-group differences in episodic memory, working memory, or accuracy in attention. Based on the results of the BRIEF-A, a self-report measure of executive function, Memophenol™ intake was associated with a greater improvement in the global score. This was specifically characterized by a greater improvement in the Metacognition index. However, there were no between-group differences in changes in other self-report measures of cognitive abilities or quality of life. Memophenol™ intake was well tolerated, and although there was a trend suggesting a greater frequency of mild digestive-related adverse reactions in the Memophenol™ group, no participant withdrew from the study due to reported adverse effects from treatment. In fact, study dropouts were more common in the placebo group, with many participants citing adverse reactions from the capsules as the reason for withdrawal from the study.

Due to significant differences in the populations recruited, doses administered, delivery forms administered, outcome measures used, and treatment periods, the results of this study are difficult to compare to previously completed trials on the cognitive-enhancing effects of grape and/or blueberry administration. In recent systematic reviews examining the impact of grape (Bird et al., 2022) and blueberry (Hein et al., 2019) administration on cognitive performance, it was concluded that there was some evidence of efficacy. Moreover, in a systematic review of berry-based supplements and foods, it was reported that they may have beneficial effects on resting brain perfusion, cognitive function, memory performance, executive functioning, processing speed, and attention indices (Bonyadi et al., 2022). However, substantial differences in the clinical trials make robust conclusions difficult. Previous trials with older adults experiencing MCI or subjective memory complaints have demonstrated that compared to a placebo, grape supplementation for 12 weeks was associated with better performance in overall cognitive performance, attention, language, and immediate and delayed memory (Calapai et al., 2017), and the 3-month intake of Concord grape juice improved verbal learning (Krikorian et al., 2010). Clinical trials on blueberry supplementation in adults with MCI or older adults with subjective memory complaints have identified improvements in memory interference and self-reports of memory encoding difficulties in everyday life after 12 weeks (Krikorian et al., 2022); improved memory discrimination and fewer self-reported cognitive symptoms after 24 weeks (Mcnamara et al., 2018); fewer repetition errors in a verbal learning task after 12 weeks (Miller et al., 2018); and improvements in the speed of processing after 6 months of intervention (Cheatham et al., 2022).

Despite no change in episodic memory (primary outcome measure), working memory, or accuracy of attention, Memophenol™ was associated with improvements in reaction time/speed of information processing, and visuospatial learning (measured using the location learning task). It is important to note that the greater improvement in reaction time/speed of information processing in the Memophenol™ group was primarily due to a statistically significant between-group difference in the reaction time for the picture recognition task and, to a lesser extent, a trend of between-group differences on the reaction time for the word recognition task. In three out of the six tasks that provided a collective measure of the speed of information processing, there was no statistically significant improvement in the Memophenol™ group over time. Therefore, future trials should examine further the effects of Memophenol™ on the speed of information processing.

Overall, these findings may have important implications for the progression of MCI. In a meta-analysis of 7 studies, it was confirmed that reaction time is slower in people with MCI, and its slowing may be an early sign of AD (Andriuta et al., 2019). Moreover, it has been demonstrated that performance on the location learning task is worse in people with AD compared with MCI, indicating that this measure could discriminate between MCI and AD (Kessels et al., 2010). However, examining the efficacy of Memophenol™ in slowing the progression of MCI and its transition into AD requires longer trials.

Compared to the placebo, Memophenol™ was associated with a larger improvement in the BRIEF-A global score, particularly on the Metacognition index. The BRIEF-A Metacognition index refers to one’s ability to initiate, plan, organize, self-monitor, and sustain working memory. It provides a self-assessment of one’s ability to cognitively self-manage tasks and monitor performance. In a study on the BRIEF-A, adults with MCI and subjective cognitive complaints reported significant difficulties with selective aspects of executive functioning relative to healthy controls despite clinically normal performance on several neuropsychological tests of executive function. These findings suggest that the BRIEF-A may be sensitive to subtle changes in executive function (Rabin et al., 2006). Although not measured in this study, the informant-reported BRIEF-A also seems to provide a reliable indicator of cognitive deficits in older adults (Scholz and Donders, 2022).

It is important to note that in this study, several statistically significant improvements were identified in the placebo group over time. This was demonstrated by improvements in immediate and delayed word recall, choice reaction time, and reaction time on the Stroop task. Moreover, there was a statistically significant reduction in the CFQ score over time in the placebo group, but no significant changes were observed in the remaining self-report measures (BRIEF-A and CASP). Improvements in the computer-based cognitive tasks may be partly due to practice effects; however, for the placebo group, this was only observed in 4 of the 16 administered tasks, compared with statistically significant, or near significant, improvements in 8 cognitive tasks in the Memophenol™ group. These results suggest that practice and placebo responses partly accounted for changes observed over time in participants, but this did not occur in all tasks. Some of the practice effects may have been minimized in the study as computer-based tasks were only completed on 3 occasions over a 6-month period. Additionally, it is possible that the re-administration of the cognitive tasks masked some of the performance deteriorations that might be observed in people with MCI over time.

How grapes and blueberries affect cognitive functioning requires further investigation, although several mechanisms are speculated. In preclinical and *in vitro* trials, blueberry supplementation reversed neuronal aging attributed to a reduction in oxidative stress (Joseph et al., 1999); upregulated neuronal signaling proteins by elevating hippocampal levels of cAMP-response element-binding protein (CREB), extracellular signal-related kinase, and brain-derived neurotrophic factor (BDNF) (Williams et al., 2008; Rendeiro et al., 2012); and increased the viability and proliferation of adult hippocampal human neural progenitor cells (Zheng et al., 2022). Blueberries may also lower neuroinflammation (Shukitt-Hale et al., 2008) and protect against amyloid β-induced cytotoxicity (Jeong et al., 2013). Preclinical trials have also demonstrated that grapes can alleviate age-related reduction of hippocampal neurogenesis and synaptogenesis (Rastegar-Moghaddam et al., 2022); inhibit the mRNA expression of amyloid precursor protein and tau (Rapaka et al., 2019); and enhance the protein expression of p-CREB, BDNF and synaptotagmin-1 (Ma et al., 2018). Animal studies examining the effects of Memophenol™ revealed that its administration to middle-aged mice ameliorated age-induced deficits in spatial navigation learning, modulated neuroplasticity, and increased the expression of nerve growth neurotrophic factor mRNA levels (Bensalem et al., 2016, 2018). All in all, these studies demonstrate that blueberries and grapes may provide cognitive benefits *via* their antioxidant, anti-inflammatory, and neuroprotective effects; upregulation of neuronal signaling proteins; and stimulation of neurogenesis. However, as these biological actions were not investigated in this study, these mechanisms remain speculative.

### Study limitations and directions for future research

Despite some positive results from the study, further research is required to validate and expand upon the current findings. More extended treatment periods will help to identify the efficacy of Memophenol™ to reverse or slow the progression of MCI. Moreover, identifying populations that may realize greater benefits from supplementation will be important. Possibilities include younger people, adults with early and mild MCI or subjective memory complaints, or people consuming a diet low in polyphenols. Even though in this study, the habitual intake of dietary polyphenols did not influence treatment outcomes, more comprehensive dietary assessments may be required to provide a more valid measure of long-term dietary intake. Objective outcome measures and more comprehensive and sensitive neuropsychological assessments will also help to substantiate the results from self-report and computer-based cognitive tasks. These include measuring blood markers of oxidative stress, inflammation, and neurogenesis, and neural imaging to identify changes in brain centers associated with memory and cognitive performance. A more comprehensive assessment of MCI will also be important, as MCI in this study was diagnosed using the telephone version of the MoCA. Finally, further investigations are required to identify optimal treatment doses, the comparative efficacy of grapes and/or blueberries delivered in isolation, and comparative changes in cognitive performance after dietary interventions as opposed to polyphenol supplementation.

In summary, supplementation with 300 mg of a grape and blueberry extract (Memophenol™) in people with MCI for 24 weeks was associated with improvements in the speed of information processing, visuospatial learning, and self-reported executive functions. However, no between-group differences in other cognitive domains were identified, including episodic memory (the primary outcome measure). The preliminary positive results of this unique polyphenol-rich extract require further investigation in robust clinical trials.

## Data availability statement

The raw data supporting the conclusions of this article will be made available by the authors, without undue reservation.

## Ethics statement

The studies involving human participants were reviewed and approved by National Institute of Integrative Medicine Human Research Ethics Committee. The patients/participants provided their written informed consent to participate in this study.

## Author contributions

DG, CP, LP, and AL designed the research. AL and SS conducted the research. AL and PD analyzed the data. AL, SS, CP, LP, DG, VP, and PD were involved in writing the paper. All authors contributed to the article and approved the submitted version.

## Funding

This study received funding from Activ’Inside. The funder was not involved in data collection, interpretation of data, or the decision to submit it for publication.

## Conflict of interest

AL is the managing director of Clinical Research Australia, a contract research organization that has received research funding from nutraceutical companies. AL has also received presentation honoraria from nutraceutical companies. SS is an employee of Clinical Research Australia and declares no other conflicts of interest. PD and VP declare no conflicts of interest. DG, CP, and LP are employed at Activ’Inside and provided the Memophenol™ and placebo capsules.

## Publisher’s note

All claims expressed in this article are solely those of the authors and do not necessarily represent those of their affiliated organizations, or those of the publisher, the editors and the reviewers. Any product that may be evaluated in this article, or claim that may be made by its manufacturer, is not guaranteed or endorsed by the publisher.

## Footnotes

^1^ http://www.randomization.com

^2^ www.nutrilog-online.com
