# Supplementary material for: Effects of a polyphenol-rich grape and blueberry extract (Memophenol™) on cognitive function in older adults with mild cognitive impairment: A randomized, double-blind, placebo-controlled study
Source: Front Psychol. 2023 Mar 29;14:1144231. doi: 10.3389/fpsyg.2023.1144231 (PMC10095830; doi:10.3389/fpsyg.2023.1144231)
Supplement: Supplementary file 1 [file Data_Sheet_1.docx]

***Supplementary Material***

Effects of a polyphenol-rich grape and blueberry extract (Memophenol^TM^) on cognitive function in older adults with mild cognitive impairment: a randomized, double-blind, placebo-controlled study

*Adrian L Lopresti, PhD^1,2^, Stephen J Smith, MA^1,2^, Camille Pouchieu, PhD^3^, Line Pourtau, PhD^3^, David Gaudout^3^, Véronique Pallet, PhD^4^, Peter D Drummond, PhD^2^

^1^Clinical Research Australia, Perth, Western Australia, 6023, Australia

^2^Healthy Ageing Research Centre and Discipline of Psychology, College of Science, Health, Engineering and Education, Murdoch University, Perth, Western Australia, 6150, Australia

^3^Activ'Inside, F-33750 Beychac-et-Caillau, France.

^4^Université de Bordeaux, INRAE, Bordeaux INP, NutriNeurO, UMR 1286, F-33000 Bordeaux, France.

**Correspondence:**

E: [adrian@clinicalresearch.com.au](mailto:adrian@clinicalresearch.com.au)

##### Supplementary Table 1: COMPASS Tasks and order of presentation

| 1. Word presentation |
| --- |
| 1. Immediate word recall |
| 1. Picture presentation |
| 1. Computerized location learning |
| 1. Simple reaction time |
| 1. Digit vigilance |
| 1. Choice reaction time |
| 1. Numeric working memory |
| 1. Corsi Blocks |
| 1. Stroop |
| 1. Delayed word recall |
| 1. Delayed word recognition |
| 1. Delayed picture recognition |
| 1. Delayed location recognition |

##### Supplementary Table 2: COMPASS tasks used in the calculation of cognitive skills

| **Cognitive Skills** | **Tasks used in calculations** |
| --- | --- |
| Episodic memory (mean percentage of cognitive tasks) | 1. Immediate word recall (percentage correct) 2. Delayed word recall (percentage correct) 3. Word recognition (percentage correct) 4. Picture recognition (percentage correct) 5. Numeric working memory (percentage correct) 6. Location learning recall (percentage accuracy) [90 (maximum score) - displacement score/ 90 (maximum score) x 100] |
| Working memory (mean percentage of cognitive tasks) | 1. Corsi blocks (percentage) [span score/ 15^#^ x 100] 2. Numeric working memory (percentage correct)   ^#^ 15 = maximum sequence |
| Speed of information processing (mean reaction time of cognitive tasks) | 1. Simple reaction time (reaction time in milliseconds) 2. Choice reaction time (reaction time in milliseconds of correct responses) 3. Numeric working memory (reaction time in milliseconds of correct responses) 4. Picture recognition (reaction time in milliseconds of correct responses) 5. Word recognition (reaction time in milliseconds of correct responses) 6. Digit vigilance (reaction time in milliseconds of correct responses) 7. Stroop (reaction time in milliseconds of correct responses) |
| Accuracy of attention (mean percentage of cognitive tasks) | 1. Choice reaction time (percentage correct) 2. Digit vigilance (percentage correct) |
| Visuospatial learning | 1. Location learning task (displacement score during 5 trials) |

##### Supplementary Table 3. Change in COMPASS tasks (estimated marginal means)

|  |  | Memophenol^TM^ (n=64) | | | | Placebo (n=56) | | | | p-value^b^ |
| --- | --- | --- | --- | --- | --- | --- | --- | --- | --- | --- |
|  |  | Week 0 | Week 12 | Week 24 | p-value^a^ | Week 0 | Week 12 | Week 24 | p-value^a^ |  |
| Immediate word recall (% correct) | Mean | 27.17 | 29.90 | 30.80 | .018 | 27.25 | 31.11 | 32.21 | .001 | .838 |
|  | SE | 1.31 | 1.28 | 1.35 |  | 1.23 | 1.41 | 1.38 |  |  |
| Simple reaction time (in ms) | Mean | 394.96 | 354.60 | 347.73 | .059 | 363.56 | 351.32 | 357.00 | .733 | .396 |
|  | SE | 22.74 | 10.77 | 9.27 |  | 15.71 | 11.61 | 16.79 |  |  |
| Digit Vigilance (% correct) | Mean | 89.73 | 90.96 | 90.69 | .471 | 89.75 | 90.96 | 89.62 | .946 | .853 |
|  | SE | 1.21 | 1.50 | 1.52 |  | 1.45 | 1.57 | 2.53 |  |  |
| Digit Vigilance (reaction time for correct responses in ms) | Mean | 477.89 | 481.53 | 478.71 | .778 | 477.44 | 478.63 | 480.22 | .357 | .480 |
|  | SE | 4.15 | 3.85 | 3.97 |  | 4.91 | 4.77 | 4.97 |  |  |
| Choice reaction time (% correct) | Mean | 97.21 | 98.36 | 98.62 | .291 | 98.00 | 98.22 | 98.20 | .515 | .575 |
|  | SE | 1.12 | 0.32 | 0.32 |  | 0.27 | 0.29 | 0.35 |  |  |
| Choice reaction time (reaction time for correct responses in ms) | Mean | 620.61 | 578.10 | 578.81 | .021 | 606.34 | 573.70 | 579.44 | .029 | .778 |
|  | SE | 16.55 | 11.57 | 10.56 |  | 14.59 | 11.73 | 13.09 |  |  |
| Numeric Working Memory (% correct) | Mean | 94.07 | 95.16 | 94.56 | .571 | 93.26 | 94.01 | 94.49 | .248 | .642 |
|  | SE | 0.87 | 0.68 | 0.88 |  | 0.90 | 1.00 | 1.15 |  |  |
| Numeric Working Memory (reaction time for correct responses in ms) | Mean | 1177.73 | 1126.00 | 1128.88 | .072 | 1129.96 | 1099.08 | 1096.99 | .325 | .835 |
|  | SE | 36.89 | 33.15 | 31.93 |  | 30.86 | 32.51 | 42.12 |  |  |
| Corsi Blocks (span score) | Mean | 5.18 | 5.38 | 5.29 | .444 | 5.26 | 5.23 | 5.33 | .661 | .587 |
|  | SE | 0.14 | 0.13 | 0.14 |  | 0.14 | 0.15 | 0.15 |  |  |
| Stroop (% correct) | Mean | 97.07 | 97.02 | 98.34 | .404 | 95.49 | 96.18 | 97.29 | .308 | .941 |
|  | SE | 1.05 | 1.17 | 1.13 |  | 1.57 | 1.36 | 1.36 |  |  |
| Stroop (reaction time for correct responses in ms) | Mean | 1370.49 | 1274.91 | 1234.91 | .001 | 1353.34 | 1308.80 | 1252.87 | .001 | .594 |
|  | SE | 42.25 | 38.58 | 32.65 |  | 36.39 | 39.57 | 34.48 |  |  |
| Delayed word recall (% correct) | Mean | 16.86 | 18.50 | 21.60 | .002 | 15.42 | 18.87 | 20.92 | < .001 | .611 |
|  | SE | 1.26 | 1.09 | 1.33 |  | 0.99 | 1.27 | 1.30 |  |  |
| Word recognition (% correct) | Mean | 73.40 | 74.03 | 74.84 | .311 | 71.50 | 74.00 | 73.39 | .196 | .638 |
|  | SE | 1.14 | 1.17 | 1.19 |  | 1.22 | 1.30 | 1.35 |  |  |
| Word recognition (reaction time for correct responses in ms) | Mean | 1698.91 | 1554.30 | 1395.36 | .002 | 1429.51 | 1466.43 | 1353.12 | .260 | .156 |
|  | SE | 115.43 | 72.11 | 51.40 |  | 58.38 | 64.65 | 60.71 |  |  |
| Picture recognition (% correct) | Mean | 94.61 | 94.08 | 94.27 | .692 | 93.61 | 93.02 | 92.54 | .232 | .817 |
|  | SE | 0.74 | 0.87 | 0.86 |  | 0.91 | 0.96 | 1.09 |  |  |
| Picture recognition (reaction time for correct responses in ms) | Mean | 1096.76 | 1091.90 | 1042.31 | .004 | 1070.75 | 1076.57 | 1106.45 | .153 | .009 |
|  | SE | 22.60 | 25.72 | 19.38 |  | 27.60 | 25.96 | 33.71 |  |  |

Results (estimated means) are generated from generalized mixed-effects models adjusted for age, sex, BMI, educational level, dietary energy intake, and dietary polyphenol intake. ^a^P-values are generated from repeated measures generalized mixed-effects models adjusted for age, sex, BMI, educational level, dietary energy intake, and dietary polyphenol intake (time effects baseline, week 12, and 24). ^b^P-values are generated from repeated measures generalized mixed-effects models adjusted for age, sex, BMI, educational level, dietary energy intake, and dietary polyphenol intake (time x group interaction).
